# Community social capital and tooth loss in Japanese older people: a longitudinal cohort study

**DOI:** 10.1136/bmjopen-2015-010768

**Published:** 2016-04-05

**Authors:** Shihoko Koyama, Jun Aida, Masashige Saito, Naoki Kondo, Yukihiro Sato, Yusuke Matsuyama, Yukako Tani, Yuri Sasaki, Katsunori Kondo, Toshiyuki Ojima, Tatsuo Yamamoto, Toru Tsuboya, Ken Osaka

**Affiliations:** 1 Department of International and Community Oral Health, Tohoku University Graduate School of Dentistry, Miyagi, Japan; 2 Department of Social Welfare, Nihon Fukushi University, Aichi, Japan; 3 Department of Health Education and Health Sociology, School of Public Health, The University of Tokyo, Tokyo, Japan; 4 Center for Preventive Medical Sciences, Chiba University, Chiba, Japan; 5 Department of Community Health and Preventive Medicine, Hamamatsu University School of Medicine, Shizuoka, Japan; 6 Division of Dental Sociology, Department of Oral Science, Graduate School of Dentistry, Kanagawa Dental University, Yokosuka City, Kanagawa, Japan

**Keywords:** Social capital, Multilevel analysis, Tooth loss, Cohort study, Panel data

## Abstract

**Objective:**

To date, no study has prospectively examined the association between social capital (SC) in the community and oral health. The aim of this longitudinal cohort study was to examine the association between both community-level and individual-level SC and tooth loss in older Japanese people.

**Design:**

Prospective cohort study

**Setting:**

We utilised data from the Japan Gerontological Evaluation Study (JAGES) performed in 2010 and 2013 and conducted in 525 districts.

**Participants:**

The target population was restricted to non-institutionalised people aged 65 years or older. Participants included 51 280 people who responded to two surveys and who had teeth at baseline.

**Primary outcome measure:**

The primary outcome measure was loss of remaining teeth, measured by the downward change of any category of remaining teeth, between baseline and follow-up.

**Results:**

The mean age of the participants was 72.5 years (SD=5.4). During the study period, 8.2% (n=4180) lost one or more of their remaining teeth. Among three community-level SC variables obtained from factor analysis, an indicator of civic participation significantly reduced the risk of tooth loss (OR 0.93; 95% CI 0.88 to 0.99). The individual-level SC variables ‘hobby activity participation’ and ‘sports group participation’ were also associated with a reduced risk of tooth loss (OR 0.88; 95% CI 0.81 to 0.95 and OR 0.90; 95% CI 0.82 to 0.99, respectively).

**Conclusions:**

Living in a community with rich SC and individuals with good SC is associated with lower incidence of tooth loss among older Japanese people.

**Strengths and limitations of this study**

- This is the first prospective cohort study to examine the association between both community-level and individual-level social capital and tooth loss.
- This study surveyed people from 525 communities around Japan in order to gain a wider range of community contextual characteristics.
- More than 50 000 people aged 65 years or older participated in baseline and follow-up surveys.
- Despite this large sample size, the measurements rely entirely on self-reported data.

## Introduction

A higher prevalence of oral diseases causes an individual burden, but it also comes at a social cost. Untreated caries in permanent teeth represent the most prevalent condition, while severe periodontitis and untreated caries in deciduous teeth were the 6th and 10th most prevalent conditions of 291 diseases and injuries.^1^ Tooth loss often occurs as a result of these diseases; in fact, severe tooth loss was the 36th most prevalent condition.^1^ In 2010, the direct and indirect global economic burden caused by oral diseases amounted to US$442 billion.^2^ In addition, oral health affects general health and can exacerbate conditions such as cardiovascular disease,^3^ ^4^ dementia,^5^ ^6^ incidence of falls^7^ and functional disability.^8^

Widespread inequalities in oral health are observed across the globe, including Japan,^9^ and are associated with individual and social burdens. Social determinants of health are the most important cause of health inequalities.^10^ ^11^ Social capital, defined by Kawachi and Berkman^12^ as “resources that are accessed by individuals as a result of their membership of a network or a group”, is increasingly recognised as a determinant of population health as well as health inequality.^13^ ^14^ Recently, an increasing number of cross-sectional studies have demonstrated the association between social capital and resources obtained from social capital on oral health.^15–18^ In spite of a large volume of cross-sectional studies focused on social capital and oral health outcomes, few studies have used longitudinal observation with community-level social capital, rather than individual-level measurements. Owing to the possibility that a community's contextual social capital could affect the health of all its residents, it is important to study population health. Although one prospective study from the UK suggests that the change in an individual's social capital corresponds to plausible changes in an older person's life course, this study did not use community-level social capital measurements.^19^

Questions regarding the association between community-level social capital and oral health based on longitudinal studies remain unanswered. The aim of this longitudinal cohort study was to examine the association between community-level and individual-level social capital and poor oral health (a reduction in remaining teeth) in elderly Japanese people. We hypothesised that living in high community-level social capital at baseline predicts good oral health at follow-up even when adjusting for individual-level social capital.

## Methods

### Study setting

We utilised data from the Japan Gerontological Evaluation Study (JAGES). The JAGES Project investigated social, behavioural and health factors in people aged 65 years or older. The JAGES sample was restricted to people who did not already have physical or cognitive disabilities, which were defined as receiving long-term public care insurance benefits. This longitudinal study used the panel data from two surveys. The baseline survey was conducted between August 2010 and January 2012 among 141 452 older people. Self-administered questionnaires were mailed to the entire population of 10 municipalities, and in 14 municipalities, questionnaires were mailed to randomly selected members of the population, based on the official residential registers obtained from the respective municipal governments. A total of 92 272 people responded to the questionnaire (response rate=65.2%). The follow-up survey was conducted between October 2013 and December 2013. Self-administered questionnaires used for the follow-up survey were subsequently mailed to the same municipalities and respondents. Collectively, 62 438 individuals completed both the 2010 and 2013 questionnaires.

**Figure 1:**
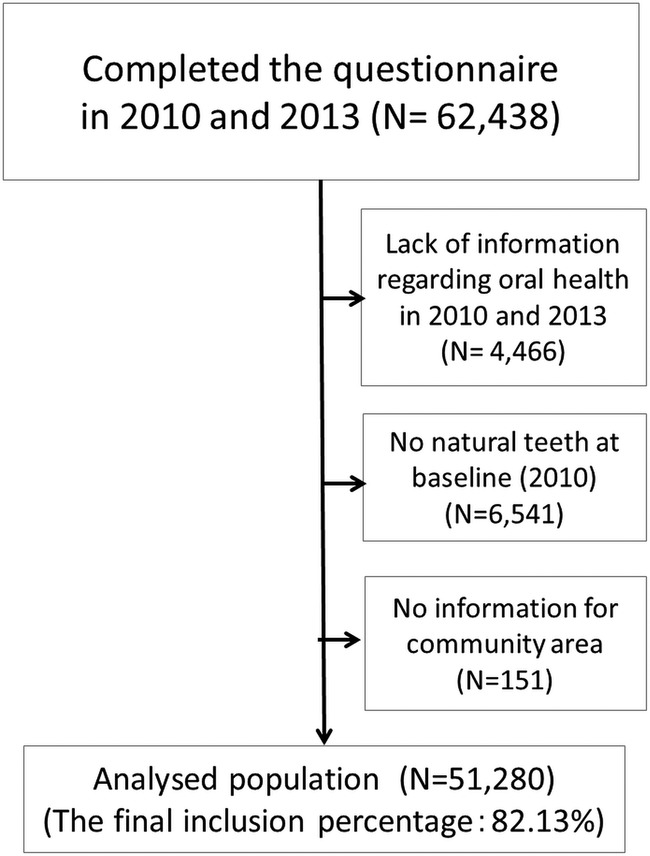
Data from 51 280 respondents were included in the analysis.

Of these respondents, 4466 were excluded because of a lack of information regarding oral health in 2010 or 2013. We excluded another 6541 individuals who had no natural teeth at baseline (2010), and 151 individuals whose information for their residential area could not be obtained. Finally, 51 280 respondents from 525 districts were included in our analyses (figure 1).

### Outcome variables

The outcome variable used was dichotomous and defined as a ‘reduction of remaining teeth or not’ between baseline and follow-up. In both surveys, we asked for the number of remaining teeth in the following categories: ‘≥20 teeth’; ‘10–19 teeth’; ‘1–9 teeth’; ‘no natural teeth’. At the follow-up survey, if respondents chose a category with a smaller number of teeth compared to that of baseline, they were defined as people who had experienced a reduction in the number of their remaining teeth during the interim period. Data on respondents who had no natural teeth at baseline were excluded from multilevel analysis.

### Predictor variables

#### Individual-level social capital

The individual-level social capital used was participation in each community activity (volunteer groups, sports groups/clubs, hobby activity group), community trust, community attachment and social support (receive emotional support, provide emotional support, receive instrumental support) in 2010.

The response categories used for community activity participation variables were ‘once or more per week’ and ‘less than once per week’. Community trust was measured by asking two yes/no questions: ‘Do you trust the people who live in your local area?’ and, ‘Do you think that it is important to be helpful to other people in your local area?’ Community attachment was measured with the yes/no question, ‘Do you have an attachment to your local area?’ Social support was measured with the following three yes/no questions: ‘Do you have someone who listens to your concerns and complaints?’ (categorised as ‘receive emotional support’); ‘Do you listen to someone else's concerns and complaints?’ (provide emotional support); and ‘Do you have someone who looks after you when you become sick?’ (receive instrumental support).

#### Community-level social capital

We presumed that respondents who lived in the same districts were exposed to the same degree of community-level social capital (Saito M, Kondo N, Aida J, *et al.* Development of an instrument for community-level social capital among Japanese older people: the JAGES Study. Submitted 2016, *J Epidemiol Community Health*). Community-level social capital variables were obtained from factor analysis. At first, rates of each individual-level social capital response in each small district were calculated. Then, using 525 small districts as the analysis unit, factor analysis was conducted and three factors were obtained: civic participation (participation in volunteer groups, sports groups and hobby activities), social cohesion (community trust and attachment) and reciprocity (received/provided emotional support; received instrumental support). Factor scores for each small district were used as community-level social capital variables.

### Covariates

As in the studies mentioned previously, the following questions regarding sociodemographic characteristics, baseline health status and risk factors for oral health were included in the analyses as covariates: age, sex, educational attainment, annual household income, comorbidity, smoking, density of dental offices, population density and number of teeth at baseline. Age was grouped into five categories: 65–69, 70–74, 75–79, 80–84 and 85 years or older. Educational attainment was categorised as follows: <6, 6–9, 10–12 and ≥13 years. Annual household income was categorised as follows: <$20 000 (<¥2 000 000), $20 000–$29 999 (¥2 000 000–¥2 999 999), $30 000–$39 999 (¥3 000 000–¥3 999 999) and ≥$40 000 (≥¥4 000 000) (US$1=¥100). Comorbidity was measured by using the yes/no question, ‘Do you receive treatment now?’. Smoking was categorised as follows: non-smoking, non-smoking now and quit more than 5 years ago, non-smoking now and quit within 4 years and smoking now. We included density of dental offices as a continuous variable in the models. Population density was categorised as follows: urban area (≥1500 people/km^2^), suburban area (1000–1500 people/km^2^) and rural area (<1000 people/km^2^).

### Data analysis

The data were analysed by multilevel logistic regression analyses. We calculated OR and 95% CI for respondents who had a reduction in the number of remaining teeth during the study period. Since 51 280 respondents lived in 525 small districts, a two-level model (community-level and individual-level) was used. We put emphasis on the theoretical importance of the covariates and included all covariates in the multivariate model. If data were missing for explanatory variables, the corresponding observations were assigned to ‘missing’ categories. The significance level was set at p<0.05. We used SPSS V.19.0 (IBM Corp, Armonk, New York, USA) for factor analysis and Stata V.13.1 (StataCorp, College Station, Texas, USA) for multilevel analysis.

### Ethical issues

JAGES respondents were informed that participation in the study was voluntary, and that completing and returning the self-administered questionnaire by mail indicated their consent for participation in the study.

## Results

Of 51 280 respondents, 23 924 men and 27 356 women were included in the analysis. The average age of the 51 280 respondents was 72.5 years (SD=5.4). Among the respondents, 8.2% (n=4180) reported a reduction in the number of their remaining teeth. Table 1 shows the descriptive statistics for each variable. Participants who were older, with less education, lower incomes, living in rural areas, with no emotional social support, having between 10 and 19 teeth, or who were smokers tended to have a higher incidence of tooth loss.

**Table 1:** Baseline characteristics of respondents and reduction of remaining teeth at follow-up (n=51280)

	Reduction of remaining teeth (N, %)				
	No		Yes		Total
Sex					
Man	21 652	91	2272	9	23 924
Woman	25 448	93	1908	7	27 356
Age (years)					
65–69	16 367	93	1187	7	17 554
70–74	14 899	92	1281	8	16 180
75–79	10 748	91	1089	9	11 837
80–84	2798	89	346	11	3144
85+	1111	88	152	12	1263
Education (years)					
<6	541	88	77	12	618
6–9	19 210	91	1923	9	21 133
10–12	16 832	93	1299	7	18 131
≥13	8957	93	702	7	9659
Annual household income					
<$10 000	4727	90	504	10	5231
$10 000–$19 999	13 758	92	1208	8	14 966
$20 000–$29 999	10 198	92	832	8	11 030
$30 000–$39 999	6472	93	502	7	6974
≥$40 000	4768	93	364	7	5132
Living area					
Urban area	12 844	93	897	7	13 741
Suburban area	22 231	92	1987	8	24 218
Rural area	12 025	90	1296	10	13 321
Hobby activity					
Less than once per week	23 139	91	2207	9	25 346
Once or more per week	16 695	93	1232	7	17 927
Sports group					
Less than once per week	28 213	92	2596	8	30 809
Once or more per week	10 384	93	756	7	11 140
Volunteer group					
Less than once per week	32 222	92	2797	8	35 019
Once or more per week	4688	92	388	8	5076
Community trust					
No	12 544	92	1098	8	13 642
Yes	32 310	92	2879	8	35 189
Community reciprocity					
No	19 386	92	1657	8	21 043
Yes	25 252	92	2294	8	27 546
Community attachment					
No	7947	92	709	8	8656
Yes	37 904	92	3356	8	41 260
Receive emotional support					
No	2305	90	268	10	2573
Yes	42 452	92	3677	8	46 129
Provide emotional support					
No	2516	90	295	10	2811
Yes	42 015	92	3622	8	45 637
Receive instrumental support					
No	2127	92	176	8	2303
Yes	42 834	92	3788	8	46 622
Number of teeth in 2010					
≥20	19 902	92	1825	8	21 727
10–19	13 775	90	1508	10	15 283
1–9	13 423	94	847	6	14 270
Smoking					
Non-smoking	26 527	93	2045	7	28 572
Non-smoking now, quit before 5 years	10 309	92	941	8	11 250
Non-smoking now, quit within 4 years	2096	90	240	10	2336
Smoking	4304	89	551	11	4855
Do you have hospital treatment?					
Yes	32 255	92	2771	8	35 026
No	11 154	91	1052	9	12 206

Table 2 shows the results of the multilevel logistic analysis. In the sex-adjusted and age-adjusted model, a significant association between community-level social capital and incidence of tooth loss was observed at two variables, ‘civic participation’ and ‘social cohesion’ (OR 0.84; 95% CI 0.80 to 0.89 and OR 1.14; 95% CI 1.08 to 1.20, respectively). Among the individual-level social capital variables, ‘hobby activity participation’ and ‘sports group participation’ were signiﬁcant for reducing the risk of tooth loss (OR 0.81; 95% CI 0.76 to 0.88 and OR 0.82 95% CI 0.76 to 0.90, respectively). When all variables were included in one model, living in a rich community-level social capital district at baseline and the incidence of tooth loss were observed at the variable ‘civic participation’ (OR 0.93; 95% CI 0.88 to 0.99). The individual-level social capital variables ‘hobby activity participation’ and ‘sports group participation’ still had signiﬁcant associations (OR 0.88; 95% CI 0.81 to 0.95 and OR 0.90 95% CI 0.82 to 0.99, respectively).

**Table 2:** Data are presented as ORs (95% CIs), p value of reduction of remaining teeth of the respondents (n=51 280)

	Sex and age adjusted analysis OR (95% CI), p value		Multivariate analysis OR (95% CI), p value	
Sex (ref woman)				
Man			0.78 (0.71 to 0.85)	<0.001
Age (ref 65–69 years)				
70–74			1.26 (1.16 to 1.37)	<0.001
75–79			1.54 (1.40 to 1.68)	<0.001
80–84			1.97 (1.73 to 2.25)	<0.001
85+			2.23 (1.85 to 2.69)	<0.001
Education (ref ≥13 years)				
<6	1.67 (1.29 to 2.16)	<0.001	1.42 (1.10 to 1.85)	0.008
6–9	1.31 (1.19 to 1.44)	<0.001	1.17 (1.06 to 1.29)	0.002
10–12	1.04 (0.95 to 1.15)	0.412	1.01 (0.91 to 1.11)	0.887
Annual household income (ref≥$40 000)				
<$10 000	1.42 (1.23 to 1.64)	<0.001	1.30 (1.12 to 1.50)	<0.001
$10 000–$19 999	1.14 (1.01 to 1.29)	0.039	1.10 (0.97 to 1.24)	0.14
$20 000–$29 999	1.05 (0.93 to 1.20)	0.426	1.04 (0.92 to 1.19)	0.514
$30 000–$39 999	1.01 (0.87 to 1.16)	0.936	1.01 (0.88 to 1.16)	0.908
Living area (ref rural area)				
Urban area	0.63 (0.57 to 0.70)	<0.001	0.69 (0.57 to 0.82)	<0.001
Suburban area	0.82 (0.76 to 0.90)	<0.001	0.87 (0.80 to 0.95)	0.001
Community-level social capital				
Civic participation	0.84 (0.80 to 0.89)	<0.001	0.93 (0.88 to 0.99)	0.017
Social cohesion	1.14 (1.08 to 1.20)	<0.001	1.05 (0.98 to 1.12)	0.135
Reciprocity or support	1.04 (0.97 to 1.11)	0.28	0.98 (0.90 to 1.05)	0.550
Density of dental office				
Density of dental office per 10 000 people	0.87 (0.84 to 0.91)	<0.001	1.01 (0.94 to 1.08)	0.876
Individual-level social capital				
Hobby activity	0.81 (0.76 to 0.88)	<0.001	0.88 (0.81 to 0.95)	0.002
Sports group	0.82 (0.76 to 0.90)	<0.001	0.90 (0.82 to 0.99)	0.027
Volunteer group	0.98 (0.88 to 1.10)	0.722	1.08 (0.96 to 1.21)	0.194
Community trust	0.98 (0.91 to 1.05)	0.58	0.99 (0.90 to 1.08)	0.760
Community reciprocity	1.03 (0.96 to 1.10)	0.377	1.06 (0.98 to 1.15)	0.152
Community attachment	0.96 (0.88 to 1.05)	0.354	0.96 (0.87 to 1.06)	0.404
Receive emotional support	0.82 (0.72 to 0.94)	0.004	0.89 (0.75 to 1.05)	0.179
Provide emotional support	0.81 (0.72 to 0.92)	0.001	0.89 (0.76 to 1.04)	0.132
Receive instrumental support	1.04 (0.89 to 1.22)	0.626	1.13 (0.96 to 1.34)	0.151
Number of teeth in 2010 (ref ≥20 teeth)				
10–19	1.14 (1.06 to 1.23)	<0.001	1.06 (0.98 to 1.14)	0.142
1–9	0.61 (0.56 to 0.66)	<0.001	0.52 (0.48 to 0.57)	<0.001
Smoking (ref non-smoking)				
Non-smoking now, quit before 5 years	0.98 (0.89 to 1.08)	0.665	1.05 (0.95 to 1.16)	0.351
Non-smoking now, quit within 4 years	1.30 (1.12 to 1.51)	0.001	1.39 (1.19 to 1.62)	<0.001
Smoking	1.48 (1.32 to 1.66)	<0.001	1.58 (1.41 to 1.77)	<0.001
Do you have hospital treatment? (ref no)				
Yes	1.17 (1.08 to 1.26)	<0.001	1.16 (1.07 to 1.25)	<0.001
Random-effects parameters				
Community-level variance Ωμ (SE)			0.0038 (0.0062)	

## Discussion

To the best of our knowledge, this is the first study to examine the association between both community-level and individual-level social capital and oral health using longitudinal data. The results suggest that living in a community with a higher density of civic participation (a measurement of community-level social capital) at baseline was associated with future low risk of tooth loss. This association was still significant even after adjusting for individual-level social participation variables that were also beneficial to oral health.

The results of the present longitudinal analysis were similar to previous cross-sectional studies. In Japan, a previous study demonstrated a significant positive association between social participation and dental health status among older people.^20^ Another cross-sectional study suggested that community-level horizontal social capital and vertical social capital have different effects on health; only the former had a contextual effect on dental status.^21^ A review of the papers on social capital and oral health also reported the beneficial association between social capital and oral health.^16^ This review, however, pointed out the need for a longitudinal analysis. The present study adds evidence supportive of an association between social capital and oral health by cohort study. In addition, those people who had 10–19 remaining teeth at baseline tended to lose their teeth (table 2). This was consistent with the results of a previous study in Japan that used data from a nationwide dental survey.^22^ Therefore, it is important to prevent tooth loss through public health interventions, individual efforts and clinical care.

There are numerous possible pathways between social capital and oral health. Rouxel *et al*^16^ summarised the hypothesised pathways linking social capital and oral health: behavioural and psychosocial, via access to oral health services and via policy development. Regarding the behavioural pathway, social capital is considered to affect health behaviours through social contagion and informal social control.^12^ As an example, one study observed the contagion of smoking cessation following a social network.^23^ Regarding the psychosocial pathway, social capital is considered to be associated with reducing psychosocial stress, a possible risk factor for oral diseases.^24^ Through collective efficacy, a community with rich social capital can establish health-promoting policies.^12^ In this context, we supposed that although population density of dental clinics was sparse during the 1960s–1970s in Japan, the establishment of a dental clinic might be promoted in a community with rich social capital. Improving access to dental care could contribute to oral health in a community because access to dental care has been reported to promote oral health.^25^

From the present results, social capital may contribute to improvements in oral health. Previous intervention studies attempted to promote health through the enhancement of social capital.^26–28^ Participation in the community salon (a resident-centred community intervention programme) contributed to the prevention of incident functional disability.^26^ ^28^ Hikichi *et al*^28^ found that participation in the community salon contributed to the prevention of incident functional disability. Although previous intervention studies related to social capital did not examine the effects on oral health, public health interventions enhancing social capital, as described above,^26–28^ might improve oral health.

The strengths of our study are its prospective cohort design and its use of panel data. This design was suitable for the inference of causality compared to previous cross-sectional studies. This is the first multilevel study of social capital and oral health using longitudinal data, including both individual-level social capital and community-level social capital. In addition, this study enabled us to consider a wider range of community contextual characteristics by surveying 525 communities in Japan with more than 50 000 older-age participants.

This study has some notable limitations. First, while this survey was large, oral health (in terms of number of remaining teeth) was self-reported and even though the validity of this measure has been well established with respect to objective measures,^29–31^ the longitudinal change of self-reported dental health was imprecise relative to clinical dental check-ups. Second, the follow-up periods differed between municipalities. Since some municipalities had shorter follow-up periods than others did, it was difficult to conclude causality in this study. Third, our study included no information about changes in social capital. Therefore, there is the possibility that time-varying, confounding factors such as economic changes or natural disasters may have biased our results. However, this study aimed to examine whether baseline social capital was associated with follow-up tooth loss in a cohort study; therefore, we applied the present cohort study design. Even if we could have used change of social capital, it is very difficult to determine causality with only two time point observations.

## Conclusion

This large-scale cohort study covered a broad area of this country and has provided evidence that high community-level and individual-level social capital at baseline is associated with a lower incidence of tooth loss at follow-up among older Japanese people.
